# Optimal Robust Motion Controller Design Using Multiobjective Genetic Algorithm

**DOI:** 10.1155/2014/978167

**Published:** 2014-05-08

**Authors:** Andrej Sarjaš, Rajko Svečko, Amor Chowdhury

**Affiliations:** ^1^Faculty of Electrical Engineering and Computer Science, University of Maribor, Smetanova 17, 2000 Maribor, Slovenia; ^2^Margento B.V., Crystal Tower, Orlyplein 10, 1043 DP Amsterdam, The Netherlands

## Abstract

This paper describes the use of a multiobjective genetic algorithm for robust motion controller design. Motion controller structure is based on a disturbance observer in an RIC framework. The RIC approach is presented in the form with internal and external feedback loops, in which an internal disturbance rejection controller and an external performance controller must be synthesised. This paper involves novel objectives for robustness and performance assessments for such an approach. Objective functions for the robustness property of RIC are based on simple even polynomials with nonnegativity conditions. Regional pole placement method is presented with the aims of controllers' structures simplification and their additional arbitrary selection. Regional pole placement involves arbitrary selection of central polynomials for both loops, with additional admissible region of the optimized pole location. Polynomial deviation between selected and optimized polynomials is measured with derived performance objective functions. A multiobjective function is composed of different unrelated criteria such as robust stability, controllers' stability, and time-performance indexes of closed loops. The design of controllers and multiobjective optimization procedure involve a set of the objectives, which are optimized simultaneously with a genetic algorithm—differential evolution.

## 1. Introduction

Modern positioning systems require high performance and complex operation, which demand highly efficient and complex controller structures. Complex controller algorithms and their designs are mostly related to very complex design procedures and optimisation techniques, with which designers try to achieve the desired performance specification. To date, many advanced design methods have been developed, based on different structures of closed-loop systems and paradigms. The most widely used designs are based on disturbance observers [1–3], internal model control [4], and model based disturbance attenuation [5]. The disturbance observer designs have been mostly used in industrial environments because of its simple transparent structure and capability to ensure proper tradeoff between robustness and performance properties. Tradeoffs between criteria can be handled with heuristic optimization approaches or mathematical programming optimization techniques, abbreviated as LP, QP, SDP, NP, and so forth [6, 7]. Optimization-based methods in computer aided design (CAD) have proved to be a valuable tool in engineering practice, with which one can achieve proper performance and suitable controllers for closed-loop systems. In general, the optimization procedure can be divided into convex or nonconvex optimization problems.

Mathematical programming approaches, such as LP, QP, and SDP, are very efficient for convex problems, which cover many modern robust controller designs in state space approach [8–10]. However, there are also many control problems, where the evaluation of the objective is not strictly convex or where the desired criteria cannot be combined into a set of convex objective functions. For example, polynomial synthesis with fixed order controller in a transfer function form and controller structure optimization is a basically nonconvex problem [11, 12]. One way to overcome this problem is by combining heuristic optimization methods with new or conventional controller designs [13–15]. This combination provides a set of efficient tools to address complex multivariable problems with performance constraints [13]. There is much evidence which indicates high efficiency of the heuristic optimization technique, especially with genetic algorithms (GAs). The GA optimization technique with well-formulated objective functions can preserve satisfactory results of controlled systems while overcoming some problems and limitations of conventional designs [13, 16–18].

This paper considers a robust disturbance observer (DOB) in a robust internal compensator framework (RIC) with multiobjective optimization of different robustness and performance criteria. The DOB structure has already been studied in detail and has several different structural approaches [1, 19]. The more convenient and straightforward methods of DOB are based on Q-filter and inverse nominal models within the internal feedback branch [1, 20, 21]. The more advanced approaches are based on internal reference models in RIC framework [2, 20]. Both methods, Q-filter and RIC, deal well with disturbance attention and have similar approaches and structures [2, 20, 22]. However, the RIC approach is more transparent and reliable in comparison with the Q-filter design. The main difference between both approaches is in the design of their internal loops [5, 22]. The significant disadvantage of the DOB with Q-filter design is the usage of an inverse nominal model within the internal feedback loop. Most mechanical systems are presented as strict proper functions, which prevents direct usage of the model inverse. The second disadvantage is the internal-loop structure with a low-pass Q-filter within a partially positive feedback loop. The property of the Q-filter lowers the closed-loop's stability and the selection is strictly empirical with some vague guidance for the first-, second-, and third-order filters [5, 21, 23, 24]. Each selection of approximated inverse function and Q-filter requires additional assessment of the closed-loop's performance and robustness. The RIC on the other hand has better structural transparency and can be much easily incorporated into the optimization procedure. Internal and external controllers can be acquired from the optimization procedure, so that the robustness of the RIC system can be ensured [25, 26].

This paper describes the design of an RIC disturbance observer with optimization of robustness and performance criteria with multiobjective differential evolution (DE) [27, 28]. The main contribution of the presented paper is a multiobjective optimization approach with simultaneous optimization of a set of criteria for inner and outer loops of the RIC structure. The used robustness and performance criteria are derived directly from the property of the norm *H*_*∞*_ and uncertainty models [29, 30]. The criteria have the form of even polynomials, where simple quasi-convex control problems arise. Robust stability of the system can be quite straightforwardly assessed, if the nonnegativity condition of the polynomial is provided. Even polynomials can be directly used in the optimization technique within genetic algorithm, without any other additional transformation. The second contribution of this paper is the design of a simple parameterized controller structure with selected central characteristic polynomial. The central characteristic polynomial has an admissible region in the stable half plain prescribed so that it ensures the expected performance and stability of the RIC system. The basic idea comes from the pole-colouring technique and regional pole assignment method [31, 32]. In comparison to similar methods, the presented approach uses objective functions of the regional pole placement technique and can be used for nonparametric uncertainties. The presented objectives are optimized with a genetic algorithm, differential evolution (DE). DE has evident advantages over other similar techniques: simple structure, fast convergence, lower space complexity, adaptive parameter settings, and so forth [33]. All design criteria within the DE algorithm are evaluated over the nonnegativity property of robustness criteria and roots calculations of the closed-loop characteristic polynomial for regional pole placement.

This paper is divided into seven sections. In Section 2, we describe the RIC disturbance observer for a positioning system.  Section 3 describes the regional pole placement for internal and external loops of the RIC system and applied parameterization of the controller structure. Thereafter, in Section 4, we describe performance and robustness properties with the metric *H*_*∞*_, based on nonnegativity assessments of the even polynomial.  Section 5 describes a multiobjective optimization approach with DE and a composite multiobjective function. After Section 5, a design example of an RIC system with multiobjective optimization results is provided in Section 6. The conclusions of the paper are summarized in Section 7.

## 2. RIC Disturbance Observer for Positioning Systems

**Figure 1:**
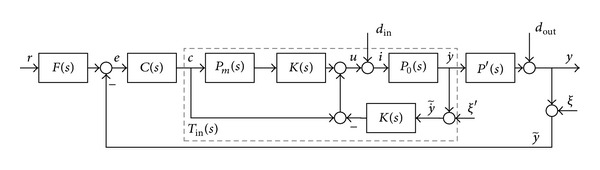
Disturbance observer in an RIC framework.

A disturbance observer in the RIC framework is composed of internal and external loops [34–36]. The design of the internal loop is based on input disturbance attention, where most often appearing disturbances are reaction and load torque, friction, and unmodeled dynamics [20, 34]. The external loop is designed so that it ensures the overall performance of the closed-loop system. The RIC structure is shown in Figure 1, where *K*(*s*), *P*_0_(*s*), *P*′(*s*), *P*_*m*_(*s*), *C*(*s*), and *F*(*s*) are the internal controller, plant, reference model, and 2 DOF structures with an external controller and a prefilter, respectively. The internal loop with *P*_0_(*s*) covers the angular velocity in relation to input torques and disturbance rejection *d*_in_, where *P*′(*s*) is the transfer function of the rotary encoder. The transfer function of the rotary encoder is mostly treated as a system with pure integral behavior. The RIC transfer functions are

(1) $$\begin{array}{c} P_{0}\left(s\right)=\frac{B_{0}\left(s\right)}{A_{0}\left(s\right)},\;\;P^{\prime }\left(s\right)=\frac{B^{\prime }\left(s\right)}{A^{\prime }\left(s\right)},\;\;K\left(s\right)=\frac{L_{K}\left(s\right)}{R_{K}\left(s\right)},\\ P_{m}\left(s\right)=\frac{B_{m}\left(s\right)}{A_{m}\left(s\right)},\;\;C\left(s\right)=\frac{L_{C}\left(s\right)}{R_{C}\left(s\right)},\;\;F\left(s\right)=\frac{L_{F}\left(s\right)}{R_{F}\left(s\right)}. \end{array}$$

The matrix form of the RIC system in Figure 1 is

(2) [F−100F−10−C(PmK+1)100K0−P0P′10000−1100−P0001](eiyy~y˙~)  =(rdindoutξξ′),

where *r*,  *d*_in_,  *d*_out_,  *ξ*, and  *ξ*′ are reference signal, input disturbance, output disturbance, internal noise, and external noise, respectively.

The closed-loop transfer function is(3)
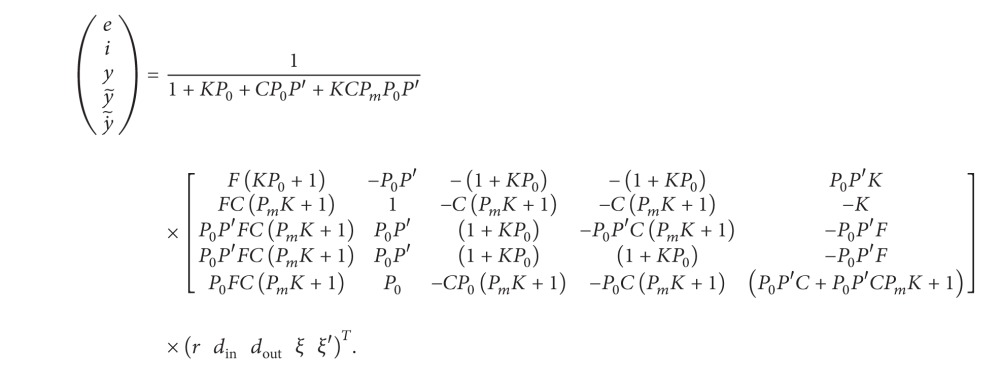



The RIC system in Figure 1 is slightly a modified classic structure. The internal loop in the modified structure embraces only the angular velocity, while the classical internal loop uses for the feedback branch the same output as for the external loop [21, 22]. The modified structure provides indirect decupling of the internal and external loops, where the poles of the internal loop do not directly influence the zeroes of the external loop. Most RIC designs include standard controller structures in internal loops [20, 24]. Standard controllers, like PID or PI, with integral behaviour improve disturbance rejection for low-frequency disturbance *d*_in_. On the other hand, internal integral behaviour causes a double-integrator effect on the external loop, which significantly lowers the stability domain and generates undesired responses on the output disturbances and reference signals. This paper will consider an RIC structure, shown in Figure 1, where the internal integral behaviour of the controller *K*(*s*) does not directly influence the external loop. For the sake of the RIC structure simplification, we assume that the prefilter function is constant and is equal to *F*(*s*) = 1.

## 3. Regional Pole Placement of the RIC System

The design procedure of the RIC system can be addressed in two steps: the first step covers the internal loop controller design and the second step the external loop controller design. Both controllers are designed with the regional pole placement technique based on the selected central polynomial in the expected stable area. The expected area is freely chosen by the designer and exhibits closed-loop dynamic performance. The parameterization of controllers is applied to ensure good disturbance rejection and robustness properties.

Let us consider the internal loop of the system in Figure 1. The closed-loop system is

(4) (y˙i)=11+KP0[P0(1+KPm)P01+KPm1](cdin),(y˙i)=Sin[P0(1+KPm)P01+KPm1](cdin),

where *S*_in_ is the sensitivity function of the internal closed-loop. The characteristic polynomial is defined as

(5) $$\begin{array}{c} A_{0}\left(s\right)R_{K}\left(s\right)+B_{0}\left(s\right)L_{K}\left(s\right)=C_{\text{in}}\left(s\right). \end{array}$$

Equation (5) is the standard starting point of pole placement design. The solution of the equation is provided under strict polynomial degree conditions [37]. The presented approach uses only the conditions when an exact solution does not exist, deg⁡*R*_*k*_ ≤ deg⁡*A*_0_ − 2. In this case, the controller structure does not depend on plant order, like in the classic pole placement design, and is arbitrarily selected by the designer. The only way to satisfy expression (5) is regional pole placement with a prescribed region and deviation assessment, as described in [32]. Deviation is assessed between the given polynomial *C*_in_(*s*) and the candidate polynomial obtained with optimization. The term prescribed region of the closed polynomial is similar to the term domain stability [29, 38]. The prescribed region with the selected central polynomial offers more leeway for further multiobjective optimization of robustness and performance criteria. The polynomial equation for regional pole placement is

(6) $$\begin{array}{c} A_{0}\left(s\right)R_{K}\left(s\right)+B_{0}\left(s\right)L_{K}\left(s\right)={\tilde{C}}_{\text{in}}\left(s\right),\;{\tilde{C}}_{\text{in}}\left(s\right)\approx C_{\text{in}}\left(s\right), \end{array}$$

where ${\tilde{C}}_{\text{in}}(s)$ is the candidate polynomial obtained with the optimization with controller coefficient *K*(*s*). The prescribed region of the desired pole location can be derived from the properties of the Laplace stability domain, *D*_in_ = {*s* = *σ* + *jω* ∈ *C* | *σ* ∈ −*Re*,  *jω* ∈ *Im*}, where ${\tilde{C}}_{\text{in}}\in D_{\text{in}}$ holds true. The possible objective functions of the prescribed region for controllers *K*(*s*), *C*(*s*) are described in the following subsections.

### 3.1. Settling-Time Objective Function

**Figure 2:**
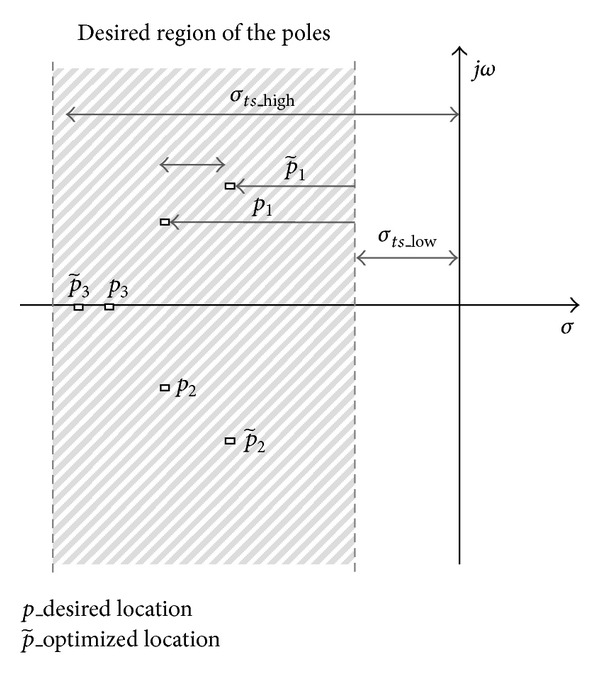
Settling-time objective function with settling-time boundaries *σ*_*ts*_low_ and *σ*_*ts*_high_.

Roughly speaking, the settling time of the closed-loop system is inversely proportional to the real part of dominant poles in the complex domain *D*_in_. The objective function can be composed so that all closed-loop poles lie in a vertical stripe, bounded by parameters *σ*_*ts*_high_ and *σ*_*ts*_low_. The vertical stripe belongs to domain *D*_in_. Parameters *σ*_*ts*_high_ and *σ*_*ts*_low_ specify the objective function criteria of the closed-loop settling time (see Figure 2).

The proposed objective function is

(7) $$\begin{array}{c} J_{1}=\underset{K}{\min}\left(\begin{array}{c} \;\;1-\underset{n}{\sum }\frac{\left|\left|Re\left\{{\tilde{C}}_{\text{in}}\right\}\right|-\left|\sigma_{ts\_\text{low}}\right|\right|}{\left|Re\left\{{\tilde{C}}_{\text{in}}\right\}\right|},\;\forall \sigma_{ts\_\text{high}}<Re\left\{{\tilde{C}}_{\text{in}}\right\}<\sigma_{ts\_\text{low}}\;\;\wedge \\ \underset{n}{\sum }\frac{\left|\left|Re\left\{C_{\text{in}}\right\}\right|-\left|Re\left\{{\tilde{C}}_{\text{in}}\right\}\right|\right|}{\max\left(\left|Re\left\{C_{\text{in}}\right\}\right|,Re\left|\left\{{\tilde{C}}_{\text{in}}\right\}\right|\right)} \end{array}\right),\;{\tilde{C}}_{\text{in}}\in D_{\text{in}}, \end{array}$$

where *σ*_*ts*_high_ and *σ*_*ts*_low_ indicate the location of the stripe boundary and *n* indicates the number of closed-loop poles. The right boundary *σ*_*ts*_high_ of the stripe prevents the possibility of obtaining poles with large negative real part values. Large negative real part pole values can cause a too high dynamic of the closed-loop system. A higher dynamic of the closed-loop can render proper real-time implementation of the discrete controller on the embedded system or cause improper behaviour of controller outputs. Improper higher dynamic of the controller output mostly manifests itself as tempestuous responses, which indicate nonoptimal energy behaviour of the closed-loop system.

The first term of the *J*_1_ indicates a normalized value of the objective if all poles lie on the left of the vertical line *σ*_*ts*_low_. The second term assesses the deviation of the real part pole value between the desired *p* and the optimized $\tilde{p}\;$ pole location, where *p* is *p* ∈ *C*_in_,  $\tilde{p}\;{\in \tilde{C}}_{\text{in}}$.

### 3.2. Rise-Time Objective Function

**Figure 3:**
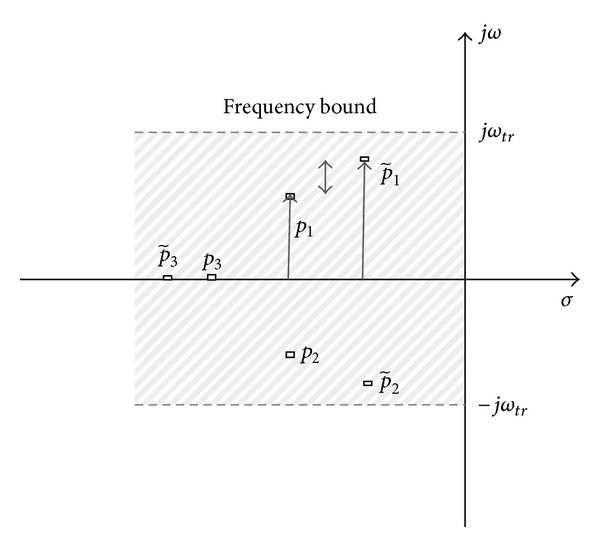
Rise-time objective function with a horizontal boundary  ± *jω*_*tr*_.

The rise time of the closed-loop system is roughly proportional to the inverse of the dominant poles imaginary values and may be upper-bounded by the parameter *ω*_*tr*_. The objective function can be composed so that all closed-loop poles lie on the horizontally bounded stripe with bounds  ± *jω*_*tr*_ (see Figure 3). The selected stripe belongs to domain *D*_in_.

The proposed rise-time objective function is

(8) $$\begin{array}{c} J_{2}=\underset{K}{\min}\left(\begin{array}{c} 1-\;\underset{n}{\sum }\frac{\left|\left|Im\left\{{\tilde{C}}_{\text{in}}\right\}\right|-\left|\omega_{tr}\right|\right|}{\left|\omega_{tr}\right|},\;\forall \left|Im\left\{{\tilde{C}}_{\text{in}}\right\}\right|<\left|\omega_{tr}\right|\\ \underset{n}{\sum }\frac{\left|\left|Im\left\{C_{\text{in}}\right\}\right|-\left|Im\left\{{\tilde{C}}_{\text{in}}\right\}\right|\right|}{\max\left(\left|Im\left\{C_{\text{in}}\right\}\right|,Im\left|\left\{{\tilde{C}}_{\text{in}}\right\}\right|\right)} \end{array}\right),\;{\tilde{C}}_{\text{in}}\in D_{\text{in}}. \end{array}$$

The objective function *J*_2_ describes the desired stripe in the complex plain, where the first term indicates the position inside the horizontal stripe and the second the imaginary deviation between the desired *p* and the optimized $\tilde{p}$ poles.

### 3.3. Damping-Ratio Objective Function

**Figure 4:**
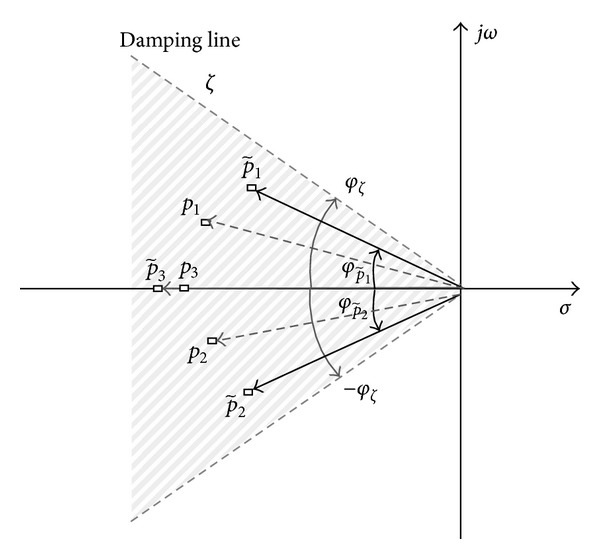
Damping-ratio objective function with parameter ± *φ*_*ζ*_.

Damping ratio is also an important design criterion of the closed-loop dynamic performance and is related to the angle of the vectors between the negative real and the imaginary axis of the dominant poles [32]. The damping ratio is determined with the angle boundary ± *φ*_*ζ*_ (see Figure 4).

The proposed damping-ratio objective function is

(9) J3=min⁡K(1−∑n||φ{C~in}|−|φς|||φς|,  ∀|φ{C~in}|<|φς|  ),  C~in∈Din.

The proposed objective function *J*_3_ represents the cone region of the desired poles. All objective functions *J*_1_–*J*_3_ represent min-optimization procedure, where all objectives are normalized on the interval [0-1].

### 3.4. The Composite Objective Function of the Dynamic Criteria

**Figure 5:**
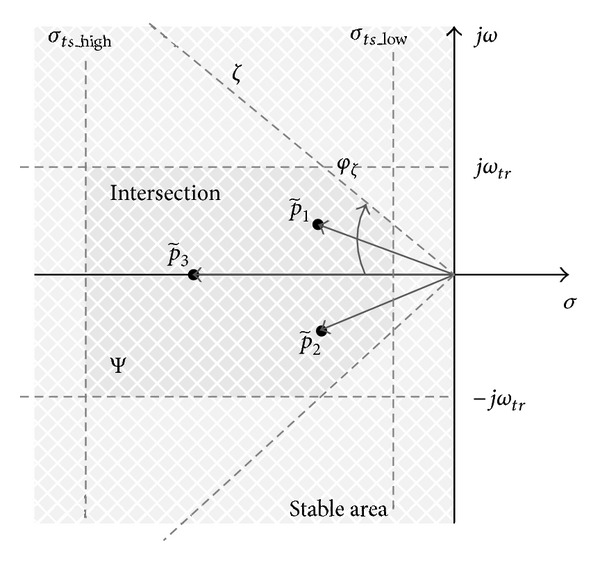
Intersection of objectives *J*_1_–*J*_3_.

The composite objective function represents the intersection of objectives *J*_1_–*J*_3_. A graphical presentation of the composite objective function is shown in Figure 5.

The composite objective function can be presented as multiobjective criteria for DE, where the expected solution area is equal to

(10) Ψin=(J1∩J2∩J3)∧min⁡⁡(dev(pin,p~in))∀Ψin∈Din,∀pin={pin∈Cin ∣ Cin∈Din},∀p~in={p~in∈C~in ∣ C~in∈Din}.

The multiobjective optimization approach will be discussed in the following sections.

### 3.5. Internal Controller Parameterization *K*(*s*)

Controller parameterization is an applied technique, which ensures satisfied properties of the closed-loop system. The internal controllers *K* and *C* can be parameterized so as to ensure good input disturbance rejection and robustness properties. In many applications, the input disturbance *d*_in_ in positioning systems represents the load torque and Coulomb's friction with a typical low-frequency characteristic [20]. The internal system can provide good elimination of low-frequency disturbances *d*_in_ and reference tracking *c* if the sensitivity function |*S*_in_| ≪ 1 has a proper damping effect on the frequency span *B* = [*ω*_*l*_, *ω*_*h*_], *c*(*B*)∧*d*_in_(*B*) ≫ 1. Under this assumption, the controller *K* can be parameterized using different polynomial structures, with a known effect on the sensitivity function |*S*_in_|. Controller parameterization with integral action *R*_*K*_(*s*) = *R*_*K*_′(*s*)*s* has a well-known influence on the sensitivity function at low-frequency characteristics, such as the known simple structures PI and PID. For the sake of operational safety, the integral action can in many cases be approximated with the stable pole (*s* + *δ*),  {*δ* ∈ *R* | 0 < *δ* ≪ 1}, where *K*(*s*) denominator polynomial is equal to *R*_*K*_′(*s*)(*s* + *δ*). In this case, the damping of the sensitivity function  *S*_in_ is lowered by parameter *δ*. The lowered damping value of  *S*_in_ is not noticeable in real-time operation, especially if the absolute damping value is lower than the absolute measurement accuracy.

A minimized tracking error *e* and good low-frequency input disturbance rejection *d*_in_ can be achieved if the following holds true:

(11) $$\begin{array}{c} \underset{\omega \to B}{\lim}\left|K\left(B\right)\right|\gg 1,\;B=\left\{B\in R\;|\;0<B\leq \omega_{\text{low}}\right\}, \end{array}$$

where *B* is the low-frequency span of the sensitivity function. Sensitivity function *S*_in_ for span *B* with controller parameterization (*s* + *δ*) is

(12) $$\begin{array}{c} \underset{\omega \to B}{\lim}\left|S_{\text{in}}\left(\delta ,\omega \right)\right|=\underset{\omega \to B}{\lim}\left(\left|S_{\text{in}}\left(\omega \right)\right|\sqrt{\omega^{2}+\delta^{2}}\right),\\ \underset{\omega \to B}{\lim}\left|S\left(B\right)\right|\approx \delta ⟹\left|S\left(\delta ,B\right)\right|\approx 0. \end{array}$$

Parameter *δ* determines the closed-loop tracking accuracy and the capability of disturbance rejection with characteristic polynomial ${\tilde{C}}_{\text{in}}(s)$. Controller structure *K* is parameterized as

(13) RK(s)={RK′(s)∏k=1p~(s+δk),RK′(s)RKpar(s).

Parameter *δ*_*k*_ represents an additional parameter for further optimization of performance and robustness criteria. The polynomial equation with parametric solutions is

(14) $$\begin{array}{c} A_{0}\left(s\right)R_{K}^{\prime }\left(s\right)\overset{\tilde{p}}{\underset{k=1}{\prod }}\left(s+\delta_{k}\right)+B_{0}\left(s\right)L_{K}\left(s\right)={\tilde{C}}_{\text{in}}\left(s\right),\\ A_{0}\left(s\right)R_{K}\left(s,\delta \right)+B_{0}\left(s\right)L_{K}\left(s\right)={\tilde{C}}_{\text{in}}\left(s\right). \end{array}$$

The solution of the optimization procedure is a proper set of polynomial coefficients, where the internal polynomial ${\tilde{C}}_{\text{in}}(s)$ belongs to the Ψ_in_ and a strong proximity condition ${\tilde{C}}_{\text{in}}(s)\approx C_{\text{in}}(s)$ holds true.

### 3.6. The Design of the External Controller *C*(*s*)

Controller *C*(*s*) design technique is the same as for the internal controller *K*(*s*), where the external characteristic polynomial ${\tilde{C}}_{\text{out}}$ is

(15) A0(s)A′(s)Am(s)RK(s)RC(s) +B0(s)LK(s)A′(s)Am(s)RC(s) +B0(s)B′(s)LC(s)Am(s)RK(s) +B0(s)B′(s)Bm(s)LK(s)LC(s)=C~out(s).

The possible controller parameterization *C*(*s*) is

(16) $$\begin{array}{c} \Gamma \left(s\right)R_{C}^{\prime }\left(s\right)\overset{\tilde{p}}{\underset{k=1}{\prod }}\left(s+\eta_{k}\right)+\Upsilon \left(s\right)L_{C}\left(s\right)={\tilde{C}}_{\text{out}}\left(s\right),\\ \Gamma \left(s\right)R_{C}\left(s,\eta \right)+\Upsilon \left(s\right)L_{C}\left(s\right)={\tilde{C}}_{\text{out}}\left(s\right), \end{array}$$

where polynomials Γ(*s*) and *Υ*(*s*) are

(17) Γ(s)=A′(s)Am(s)(A0(s)RK(s)+B0(s)LK(s)),Υ(s)=B0(s)B′(s)(Am(s)RK(s)+Bm(s)LK(s)).

Coefficient *η* can also be used as an additional parameter for criteria optimization in the same sense as parameter *δ*.

The controller *C*(*s*) is the proper solution of the optimization problem for external loop if the following conditions are satisfied:

(18) Ψout=(Jout 1∩Jout 2∩Jout 3)∧min⁡⁡(dev(pout,p~out))∀Ψout  ∈Dout,∀pout={pout∈Cout ∣ Cout∈Dout},∀p~out={p~out∈C~in ∣ C~out∈Dout}.

Objective functions *J*_out 1_–*J*_out 3_ have the same properties and meanings as objective functions *J*_1_–*J*_3_.

## 4. Nonparametric Uncertainty and Robustness Criterion of RIC

**Figure 6:**
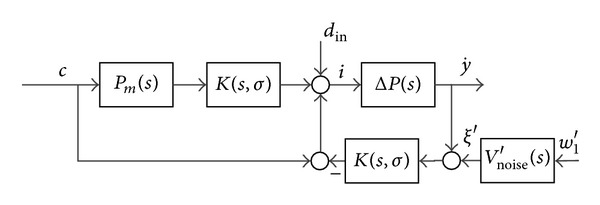
Internal loop with uncertainty models Δ*P*.

The robustness of the RIC system is assessed using uncertainty models Δ*P* and a stable proper input weight *V*_noise_′. The uncertainty models describe model deviation within the given frequency space and are represented with a nominal model and stable uncertainty weights Δ*W* [30, 39]. Weights *V*_noise_′ represent measured noise spectrum of the signal *w*_1_′. For RIC design, we assume that the reference model is *P*_*m*_ ≈ *P*_0_ ∈ *PH*_*∞*_. The internal loop of the RIC with uncertainty models is shown in Figure 6.

The robustness assessment of the internal loop should be considered with multiplicative and inverse uncertainty. The multiplicative uncertainty represents uncertainties by lower frequencies, while the inverse uncertainty represents uncertainties by higher frequencies [39].

Let us consider the multiplicative uncertainty model Δ*P*_*M*_ = *P*_0_(1 + Δ*W*_*M*_) with nominal plant *P*_0_. The closed-loop characteristic using the multiplicative uncertainty model Δ*P*_*M*_ is

(19) (y˙~i~)=(1+KP0(1+ΔWM))−1 ×[P0(1+ΔWM)(1+KPm)P0(1+ΔWM)1+KPm1](cdin).

Robust stability for the multiplicative uncertainty is preserved if the following holds true:

(20) ||ΔWMKP01+KP0||∞<1∀|Δ(ω)|<1 ω={ω∈R ∣ 0≤ω<∞},||ΔWMTin||∞<1,

where *T*_in_ is a complementary sensitivity function of the internal loop.

The internal loop with inverse uncertainty Δ*P*_*I*_ = *P*_0_(1 + Δ*W*_*I*_)^−1^ is defined as

(21) (y˙~i~)=(1+KP0(1+ΔWI)−1)−1 ×[P0(1+ΔWI)−1(1+KPm)P0(1+ΔWI)−11+KPm1] ×(cdin).

The robustness for inverse models is satisfied if the following holds true:

(22) ||ΔWI11+KP0||∞<1∀|Δ(ω)|<1 ω={ω∈R ∣ 0≤ω<∞},

where *S*_in_ is the sensitivity function of the internal loop.

The noise suppression *ξ*′ of the internal loop can be assessed with the following criterion:

(23) $$\begin{array}{c} T_{w_{1}^{\prime }}={\left(1+K\left(\delta \right)\Delta P\right)}^{-1}{V_{\text{nois}}^{\prime }}_{\text{e}}. \end{array}$$

The robustness criterion of noise suppression with the uncertainty model is

(24) $$\begin{array}{c} T_{w_{1}^{\prime }}=\;\underset{K}{\min}||\left[W_{M}\;\;W_{I}\right]\left[\begin{array}{cc} T_{\text{in}} & 0\\ 0 & S_{\text{in}} \end{array}\right]V_{\text{noise}}^{\prime }||. \end{array}$$

**Figure 7:**
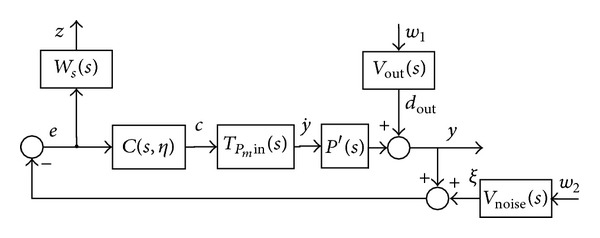
External loop optimization structure.

After derivation of robust stability conditions of the internal loop, robust stability conditions for the external loop can be presented. The purpose of external controller design is to ensure overall performance characteristics like proper reference tracking *r*, output disturbance rejection *d*_out_, and good measurement noise cancellation *ξ*. The optimization structure of the external loop is shown in Figure 7.

Selected weights *W*_*s*_, *V*_out_, *V*_noise_ belong to the PH_∞_ domain. Input weights *V*_out_, *V*_noise_ represent the input spectral characteristics of disturbance *d*_out_ and noise *ξ*, where the performance weight *W*_*s*_ is selected to ensure a smooth frequency characteristic of the external-loop sensitivity function *S*_out_(*s*). A smooth characteristic of the sensitivity function is also an additional indicator of the closed-loop time-performance characteristics.

The closed-loop characteristic of the external loop with weights *W*_*s*_, *V*_out_, *V*_noise_ is

(25) Tzw=Ws−1[(1+C(η)TinP′)−1(1+C(η)TinP′)−1(1+C(η)TinP′)−1][1VoutVnoise]    =Ws−1[Sout    SoutSout][1VoutVnoise].

The optimal solution of the optimization problem is

(26) $$\begin{array}{c} \underset{C}{\min}{||T_{zw}||}_{\infty }=\gamma_{\min}. \end{array}$$

The main goal of expression (26) is to minimize the influences of inputs *w*_1_,  *w*_2_ on the sensitivity function *S*_out_ and to ensure final feedback performance with *S*_out_ ≈ *W*_*s*_∧|*S*_out_| < |*W*_*s*_|.

### 4.1. Robustness Criteria Assessment via Nonnegativity of the Even Polynomial

Let us consider the property of the norm ||·||_*∞*_ related to robust stability with uncertainty models [30, 39]. The robust stability criterion is ensured with condition ||·||_*∞*_ < 1,**  **where the criterion is derived for a given transfer function *P*_0_(*s*) = *B*(*s*)*A* ^−1^(*s*). The condition ||*P*_0_||_*∞*_ < 1 holds if the even polynomial *π*(*ω* ^2^) is a strictly nonnegative function,

(27) $$\begin{array}{c} A\left(\omega^{2}\right)-B\left(\omega^{2}\right)=\pi \left(\omega^{2}\right)>0. \end{array}$$

The property of the function *π*(*ω* ^2^) is derived from the function Φ(*jω*), defined as in [30]:

(28) $$\begin{array}{c} \Phi \left(j\omega \right)=\gamma^{2}I-\frac{B\left(j\omega \right)}{A\left(j\omega \right)}\frac{B\left(-j\omega \right)}{A\left(-j\omega \right)}. \end{array}$$

Function Φ(*jω*) is a strictly continuous function for all ∈*R* ∪ {*∞*} and has no imaginary zeroes. The norm of the function equals ||*P*_0_|| ≤ *γ* only if Φ(*jω*) > 0 for all *ω* ∈ *R*. The robustness property for nonparametric uncertainty can be assessed with even polynomial *π*(*ω* ^2^) > 0 (27), where we assume that *γ* = 1 and *A*(*jω*)*A*(−*jω*) = *A*(*ω* ^2^), *B*(*jω*)*B*(−*jω*) = *B*(*ω* ^2^) holds true.

**Theorem 1**

The norm ||·||_*∞*_ of the system with polynomials  *A*,  *B* is ||*B*/*A*||_*∞*_ < 1  only if the corresponding polynomial *π*(*ω* ^2^), for all *ω*, is a strictly nonnegative function and *B*(*s*)*A*(*s*)^−1^ belongs to *PH*_*∞*_.

**Proof**

The simple explanation of the norm ||·||_*∞*_ is ||*B*/*A*||_*∞*_ : = sup⁡_*ω*_|*B*(*jω*)*A* ^−1^(*jω*)|, where we assume that *B*(*s*)*A*(*s*)^−1^ belongs to *PH*_*∞*_. The norm of the transfer function is ||*B*/*A*||_*∞*_ < 1 only if the ratio of polynomials |*B*(*jω*)||*A*(*jω*)|^−1^ < 1. It is obvious from the above-mentioned that the difference (27), *π*(*ω* ^2^), must be a strictly nonnegative function and that *π*(*ω* ^2^) has no real zeroes. The positivity condition of the *π*(*ω* ^2^) is an objective of robustness assessment.

Based on condition (27), the controllers' robustness property can be achieved with the assessment of the nonnegativity of the even polynomial. Each single robustness criterion ((20), (22), (24), and (26)) can be presented in the form of an even polynomial and a nonnegativity condition.

### 4.2. Robust Stability Assessment with a Nonnegativity Condition for the Internal Loop

The internal-loop robustness conditions are presented with expressions (20), (22), and (24). Based on condition (27), the even polynomial *π*_*M*_(*ω* ^2^, *δ*) for multiplicative uncertainty (20) is

(29) πM(s,δ)=C~in(s)C~in(−s)waM(s)waM(−s)−B0(s)B0(−s)LK(s,δ)LK(−s,δ)wbM(s)×wbM(−s),πM(ω2,δ)=C~inwaM(ω2)−B0LKwbM(ω2,δ)>0,

where the weight  *W*_*M*_(*s*) is  *W*_*M*_(*s*) = *w*_*bM*_(*s*)*w*_*aM*_^−1^(*s*).

Even polynomial *π*_*I*_(*ω* ^2^, *δ*) for robust stability with inverse uncertainty model (22) with stable weight *W*_*I*_(*s*) = *w*_*bI*_(*s*)*w*_*aI*_^−1^(*s*) is

(30) πI(s,δ)=C~in(s)C~in(−s)waI(s)waI(−s)−A0(s)A0(−s)RK(s,δ)RK(−s,δ)wbI(s)×wbI(−s),πI(ω2,δ)=C~inwaI(ω2)−A0RKwbI(ω2,δ)>0.

Even polynomial *π*_*T*_*w*_1_′__(*ω* ^2^, *δ*) for condition (24) with stability weights *W*_*M*_(*s*) and  *W*_*I*_(*s*) and input weight *V*_noise_′(*s*) = *v*_*b*noise_′(*s*)*v*_*a*noise_^′−1^(*s*) is

(31) πTw1M′(ω2,δ)=waMC~invanoise′(ω2)−wbMA0AmRKvbnoise′(ω2,δ),πTw1′S(ω2,δ)=waSC~invaout′(ω2)−wbSA0AmRKvbout′(ω2,δ),πTw1′(ω2,δ)=0.5(πTw1M′(ω2,δ)+πTw1S′(ω2,δ))>0.

To simplify the optimization procedure in comparison to the classic approach, the composed condition (24) is treated similarly as the criterion in augments plant transformation in a classic *H*_*∞*_ design, wherein the condition *π*_*T*_*w*_1_′__(*ω* ^2^, *δ*) (31) is a simple even polynomial function. In the multiobjective optimization approach, the condition *π*_*T*_*w*_1_′__(*ω* ^2^, *δ*) (31) can be also considered as two distinct functions: *π*_*T*_*w*_1*M*_′__(*ω* ^2^, *δ*) and *π*_*T*_*w*_1_′*S*__(*ω* ^2^, *δ*).

### 4.3. Robust Stability Assessment with a Nonnegativity Condition for the External Loop

A polynomial function for robustness and performance condition (26) for the external loop in Figure 7 can be formulated the same way as conditions (24) and (31). The even polynomial of condition (26) with performance weights *W*_*S*_(*s*) = *w*_*bS*_(*s*)*w*_*aS*_^−1^(*s*), *V*_out_(*s*) = *v*_*b*out_(*s*)*v*_*a*out_^−1^(*s*), and *V*_noise_(*s*) = *v*_*b*noise_(*s*)*v*_*a*noise_^−1^(*s*) is

(32) πw1z(ω2,η)=waSCout(ω2)−wbSA0AmA′RKRC(ω2,η),πw2z(ω2,η)=waSCoutvaout(ω2)−wbSA0AmA′RKRCvbout(ω2,η),πzw(ω2,η)=(πw1z(ω2,η)2+πw2z(ω2,η)2)>0.

As with condition (31), we can use composite objective function *π*_*zw*_(*ω* ^2^, *η*) to simplify the optimization procedure.

Additional criteria are imposed to achieve strong stability of the RIC, where the real-time operational safety for the controlled system must be preserved in case some parts of the feedback system fail, for example, sensors' failure, electronic driver failure, and faulty motor. Controllers' stability can be assessed over strict positive realness (SPR) criteria [40]. Controllers *K*(*s*),  *C*(*s*) are stable transfer functions if SPR's conditions hold true:

(33) $$\begin{array}{c} Re\left[K\left(j\omega \right)+K\left(-j\omega \right)\right]>0,\\ Re\left[C\left(j\omega \right)+C\left(-j\omega \right)\right]>0, \end{array}$$

where *Re*[*K*(*jω*)] and *Re*[*C*(*jω*)] are even functions. The even polynomials of stability conditions (33) are

(34) πKSPR(ω2,σ) =2(RK(−jω,σ)LK(jω)+RK(jω,σ)LK(−jω))>0,πCSPR(ω2,η) =2(RC(−jω,η)LC(jω)+RC(jω,η)LC(−jω))>0.

The robust conditions and the stability property of controllers (29)–(32), (34) can be directly used in a multiobjective optimization algorithm with DE.

## 5. Multiobjective Optimization with DE for RIC Design 

The paper presents a multiobjective optimization procedure with DE. Genetic algorithm DE is known as a very efficient and powerful stochastic optimization procedure. DE includes similar operation steps as other GAs, but, in comparison with other classic GAs, DE deviates the current population with scaled differences between two randomly selected members [28]. There are many reasons for using the DE algorithm for solving various optimization problems in different scientific disciplines. Compared to other similar algorithms, DE has a simpler structure and is easier to implement on real problems. Because of its simple structure, it is suitable for large scale optimization problems. The control parameters of DE have been well studied, and their influence on the optimization procedure is well known.

This paper presents the design of a robust, efficient, simple, and structured disturbance observer, based on simple objective functions for performance and robustness assessment. The main problem in optimal control design is formulating proper efficient objective functions, which can be further used in a corresponding optimization algorithm. Objective functions with their properties must ensure optimal or suboptimal solutions of the given problem. The given optimization problem in RIC structure with regional pole placement technique does not provide a convex set of objective functions. There also do not exist general straightforward procedures to convert such objectives to convex problem, which are mostly in conflict with each other and unrelated. For example, unrelated criteria are strong stability and robustness, closed-loop pole location and controller stability, controller structure and robustness performance, and so forth. In such cases where engineering simplifications are needed, it is very appropriate to use a metaheuristic optimization tool, like DE. For this reason, this paper does not deal with the efficiency of multiobjective DE algorithms and their variants but only considers the efficiency of the used approach in an optimal control problem in an RIC framework with formed objective functions for dynamic properties (7)–(9) and robustness (29)–(32) and (34).

Multiobjective optimization problems with DE involve multiple objectives, which must be optimized simultaneously, and a set of possible solutions must be obtained. The set of possible solutions is evaluated based on the concept of dominance and Pareto optimality [14, 17, 33]. The presented RIC design uses DEMO algorithm presented by Robič and Filipič [42]. The DEMO combines the advantages of DE with the mechanisms of Pareto-based ranking and crowding distance sorting. The advantage of the DEMO algorithm is that it ensures convergence to the Pareto-front and a uniform spread of individuals along the front [42].

A multiobjective function is composed of derived objective functions (7)–(9), (29)–(32), and (34) and is equal to

**Figure 8:**
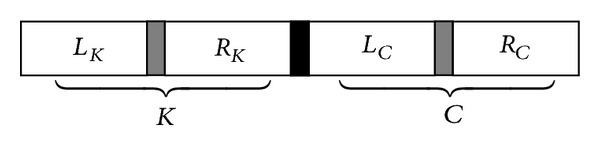
Structure of decision variable *X*.

(35) min⁡ω2∨X⁡ f(ω2,X)=[J1(X)∧Jout 1(X)J2(X)∧Jout 2(X)J3(X)∧Jout 3(X)  πM(ω2,X)  πI(ω2,X)  πTw1′(ω2,X)  πzw(ω2,X)    πKSPR(ω2,X)        πCSPR(ω2,X)    ],s.t.  f(ω2,X)>0,X∈P,

where *P* is parameter space and *X* is decision vector. Decision vector *X* contains the coefficients of polynomials  *L*_*K*_,  *R*_*K*_,  *L*_*C*_,  *R*_*C*_  (1) with possible parameterization coefficients *δ* and *η*.  Figure 8 shows the structure of the decision variable *X*.

*Note.* The solution *X* ∈ *P* is Pareto-optimal if and only if there does not exist $\tilde{X}\in P$ that satisfies $f\left(\omega^{2},X\right)<f(\omega^{2},\tilde{X})$.

The optimization algorithm starts with the preselected central polynomials *C*_in_, *C*_out_, where the controllers' degrees are prescribed in the same way as in the classic polynomial design (6), (16) [37]. The degree of controller  *K* is equal to the condition deg⁡*K* = deg⁡*C*_in_ − deg⁡*P*_0_, where deg⁡*C*_in_ > deg⁡*P*_0_ holds true. The degree of controller *C* can be determined the same way as for controller *K*, where deg⁡*C* = deg⁡*C*_out_ − (deg⁡*C*_in_ + deg⁡⁡*P*_*m*_+deg⁡*P*′) and deg⁡*C*_out_ ≥ (deg⁡*C*_in_ + deg⁡⁡*P*_*m*_+deg⁡*P*′) holds true. The optimization procedure is shown in Algorithm 1.

## 6. The Design Procedure for RIC Controllers 

The proposed robust motion controller design is demonstrated by an electromechanical positioning system with the parameters given in Table 1.

The parameters *J*,  *B*,  *k*_*P*_,  *R*, and *L* are rotor inertia, viscous friction, torque factor, terminal resistance, and rotor induction, respectively. To ensure simple controller structures of *K*(*s*) and *C*(*s*), we use a simplified model of the mechanical system *P*_0_(*s*). The simplification is often applicable if the plant has more strongly expressed dominant stable poles in comparison to other stable poles. The given model is

(36) $$\begin{array}{c} P_{0}\left(s\right)=\frac{\omega \left(s\right)}{i\left(s\right)}=\frac{k_{P}}{Js+B}=\frac{130.6}{s+0.667},\\ P^{\prime }\left(s\right)=\frac{\varphi \left(s\right)}{\omega \left(s\right)}=\frac{1}{s}. \end{array}$$

The output of the controller *K*(*s*) is an armature current *i*[*A*] through a magnetic coil, which is proportional to the electrical torque. To avoid additional nonlinearities, it is sensible to consider terminal voltage |*Ri* + *k*_*e*_ *ω*| < |*U*_limit_|, especially if the system is driven conventional by an H-bridge and PWM signal. The coefficient *k*_*e*_ is an electromotive force constant.

The uncertainty plant is given by

(37) $$\begin{array}{c} \Delta P\left(s\right)=\frac{\Delta k_{P}}{\Delta Js+\Delta B}, \end{array}$$

where the parameters vary in intervals according to changed operation points, friction, load, gear box, and so forth. The uncertainty parameter intervals are shown in Table 2.

The estimated uncertainty weights for the robustness criteria (20), (22), and (24) are

(38) WM(s)=1.34×s3+1.156×s2+0.32×s+0.062s3+1.57×s2+0.48×s+0.096,WI(s)=0.65×s2+0.39×s+0.083s2+0.82×s+0.17.Vnoise′(s)=0.0023×s+1.2·10−6s+980.3.

The desired requirements of the RIC systems are given in Table 3.

The reference model *P*_*m*_(*s*) is selected so as to ensure proper internal dynamic of the RIC structure, where holds *P*_*m*_(*s*) ≈ *P*_0_(*s*). The selected *P*_*m*_(*s*) is

(39) $$\begin{array}{c} P_{m}\left(s\right)=\frac{289.2}{s+1.5}. \end{array}$$

According to the RIC dynamic requirement for input and output disturbance rejection and tracking property, the additional performance weights are selected as follows:

(40) WS(s)=2.1×s2+0.46×s+0.001s2+3.98×s+0.99,Vout(s)=0.002×s+0.0012s+0.0014,Vnoise(s)=2.9·10−3×s+1.2·10−3s+1.001·103.

The controller transfer function *K*(*s*) is selected so as to ensure simple structure and maintain desirable closed-loop performance. The selected low-order structure is

(41) $$\begin{array}{c} K\left(s,{\tilde{r}}_{k0}\right)=\frac{l_{k1}s+l_{k0}}{r_{k1}s+\delta }, \end{array}$$

where the parameter *δ* represents controller parameterization (14) and the allowable desired value is given on the interval [10^−4^ − 10^−2^]. The value of *δ* ensures proper input disturbance rejection for low-frequency signals and stable approximate integral behaviour.

Accordingly, the following internal central polynomial *C*_in_(*s*) is chosen on the selected controller structure *K*(*s*) on the condition deg⁡*C*_in_ = deg⁡*K* + deg⁡*P*_0_. The internal central polynomial is

(42) $$\begin{array}{c} C_{\text{in}}\left(s\right)=s^{2}+2\times s+1. \end{array}$$

The polynomial *C*_in_(*s*) ensures proper dynamic and stability of the internal system. The selected allowed region *D*_in_ of the optimized internal loop poles ${\tilde{C}}_{\text{in}}(s)$ is presented in Table 4.

The selected controller structure *C*(*s*) is

(43) $$\begin{array}{c} C\left(s,{\tilde{r}}_{c0}\right)=\frac{l_{c2}s^{2}+l_{c1}s+l_{c0}}{r_{c2}s^{2}+r_{c1}s+\eta }. \end{array}$$

The parameter *η* represents *C*(*s*) controller parameterization in such a way that the double-integrator effect in the external loop is avoided. The admissible value is *η* > 50. According to controller structures *K*(*s*)  and  *C*(*s*) and the reference model *P*_*m*_(*s*), the external central polynomial is selected as

(44) Cout(s)=s6+13.5×s5+234×s4+1286×s3 +4171×s2+4773×s+1490,

where the following condition holds true: deg⁡*C*_out_ = deg⁡*C* + (deg⁡*C*_in_ + deg⁡⁡*P*_*m*_+deg⁡*P*′). The selected allowed region *D*_out_ of the optimized external-loop poles ${\tilde{C}}_{\text{out}}(s)$ is presented in Table 5.

**Figure 9:**
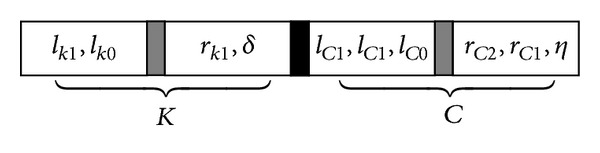
The structure of the decision variable *X* with 10 parameters.

The structure of the decision variable *X* for the given example is shown in Figure 9.

The selected parameters of DE algorithm are presented in Table 6.

## 7. Results

Optimized controllers *K*(*s*) and *C*(*s*) after optimization with DE and objective function (35) are as follows:

(45) K(s)=13.56·10−3×s+8.4·10−3s+0.18·10−3,C(s)=0.27×s2+2.3×s+2.29s2+9.324×s+102.1.

The optimization results are presented below. The difference between the selected internal central polynomial *C*_in_(*s*) and the optimized polynomial ${\tilde{C}}_{\text{in}}(s)$ is presented in Table 7.

A comparison of polynomials *C*_out_(*s*) and ${\tilde{C}}_{\text{out}}(s)$ is presented in Table 8.

From the results of polynomial comparisons in Tables 7 and 8, it is evident that the optimization procedure with Pareto-optimal solution ensures a satisfactory fitting of pole positions in the dominant region. The obtained controllers *K*(*s*) and *C*(*s*) are stable, and all poles of internal and external loops lie in the prescribed region.

The final values of robust criteria (20), (22), (24), and (26) are presented in Table 9.

**Figure 10:**
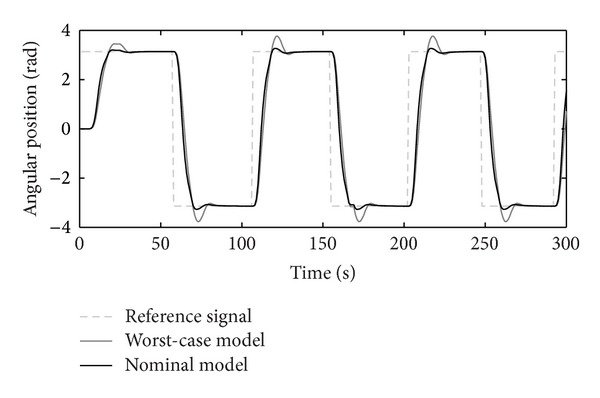
Step-reference tracking.

**Figure 11:**
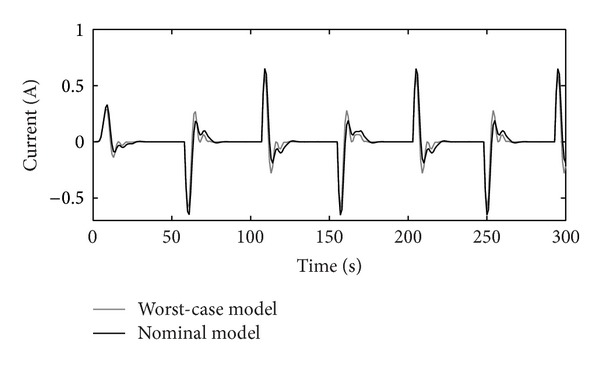
Output of the controller *K*(*s*).

**Figure 12:**
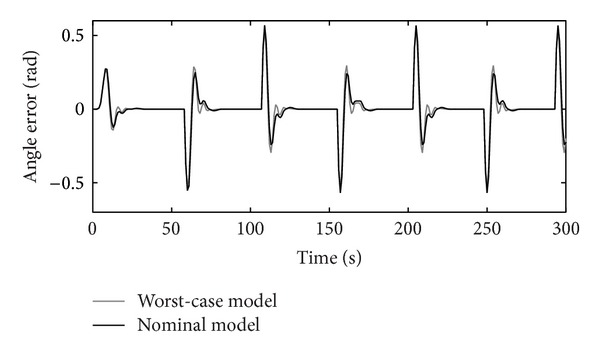
Output of the controller *C*(*s*).

**Figure 13:**
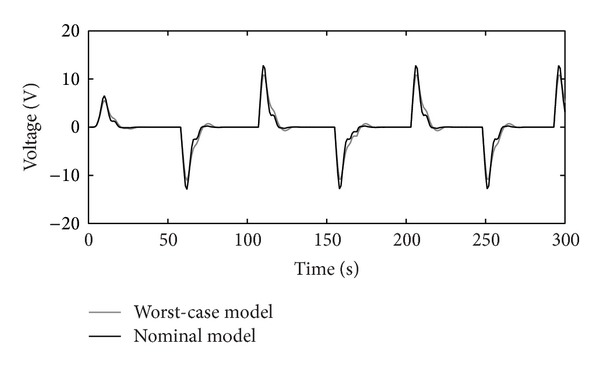
Output-voltage of the RIC system.

The tracking RIC capabilities on step-reference signals for the nominal and worst-case systems are shown in Figure 10. The reference signal presents a rotation of the RIC system for half a turn to the left and to the right. The worst-case model is selected accordingly, as a possible real operational case with values max⁡⁡(Δ*J*), max⁡⁡(Δ*B*), and min⁡⁡(Δ*k*_*P*_), chosen from Table 2. Controllers' *K* and *C* outputs are shown in Figures 11, 12, and 13, respectively.

**Figure 14:**
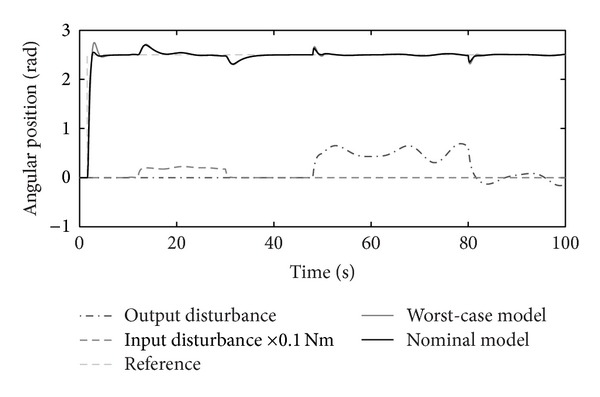
Input-output disturbance rejection.

**Figure 15:**
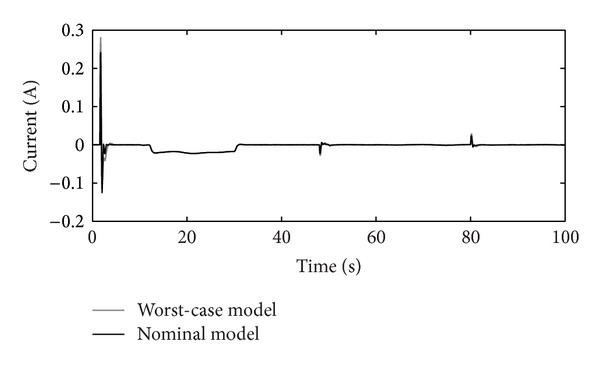
Output of the controller *K*(*s*) with disturbance attenuation.

**Figure 16:**
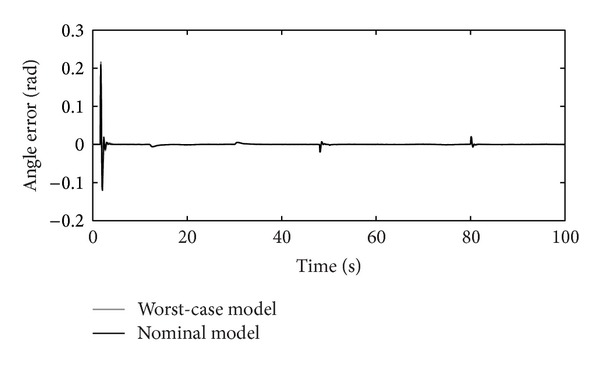
Output of the controller *C*(*s*) with disturbance attenuation.

**Figure 17:**
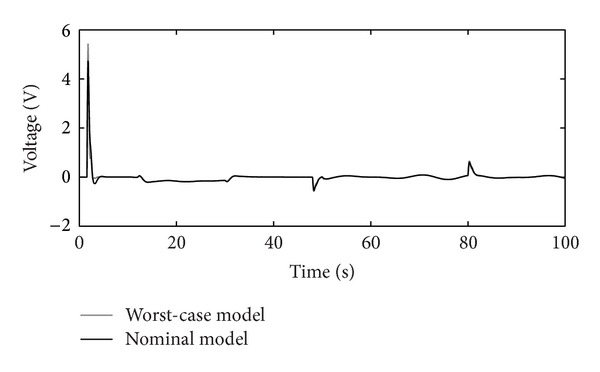
Output voltage of the RIC system with disturbance attention.

The disturbance rejection capability of the positioning system is shown in Figures 14, 15, 16, and 17.

Figures 10–17 show that the robust motion controller presented in the RIC framework satisfies all control design criteria. Table 9 and Figures 10–17 provide evidence that the stability and performance conditions are preserved. The system does not exceed the limit values for the operation voltage and current in the given operation interval, so that the system does not exhibit additional nonlinear or oscillating behaviour (Figures 11 and 13). The influence of the measured noise is also minimized with conditions ||*T*_*w*_1_′_||_*∞*_ and ||*T*_*wz*_||_*∞*_, Table 9. The system has good reference tracking and disturbance rejection within the prescribed area of system uncertainty (Figures 14–17) and simple low-order structures of the internal and external controllers.

## 8. Conclusion

This paper presented the design of a robust RIC structure for a positioning system. The proposed approach shows the capability of robustness and performance optimization over nonnegativity of an even polynomial, where regional pole placement is used. The even polynomial can be also formulated for other types of uncertainty and performance criteria. Controllers *K* and *C* can be parameterized approximately by using known characteristics, which allows the possibility of preserving strong stability of the controlled system. Optimization with the multicriterion algorithm, such as DE, offers the possibility of including many criteria and an arbitrary number of free parameters, as shown in the presented example. The criteria can include system knowledge, uncertainties, and perturbation characteristics, as well as criteria related to frequency and time domain characteristics of a closed-loop system. The presented results in the design example confirmed the validity of the proposed approach. The DE-optimization procedure with different robustness and performance criteria can be used in a wide range of different controller and feedback structures designs. The presented approach can be straightforward extended to the *H*_2_ or *H*_*∞*_/*H*_2_ controller design. Further work will be focused on the pole placement robust state space controller design for MIMO system, where the polynomial equation introduces a set of parametric solutions. In MIMO case, the exact solution of the polynomial equation is limited to the number of the inputs and outputs of the system. The parametric solutions can be used as optimization parameters, similar to the presented approach.

## Figures and Tables

**Algorithm 1:**
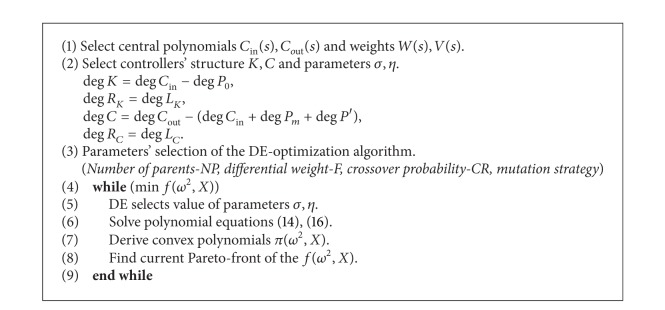
Multiobjective DE algorithm.

**Table 1:** Parameters of an electromechanical system.

Motor parameters	
*J*	6.2 · 10^−3^ kgm^2^
*B*	4.2 · 10^−3^ Nms
*k*_*P*_	0.081 Nm/A
*R*	7.9 Ω
*L*	5.7 mH

**Table 2:** Uncertainty parameters of the positioning system.

Uncertainty parameters	
Δ*J*	[2.4 ÷ 9.8] · 10^−3^ kgm^2^
Δ*B*	[3.3 ÷ 6.5] · 10^−3^ Nms
Δ*k*_*P*_	[0.6 ÷ 1.3] Nm/A

**Table 3:** RIC control requirements.

Feedback loop RIC requirements	
Settling time	*t*_*s*_ < 1.4 s
Nominal plant overshooting	*M*_Pn_ < 3%
Worst-case plant overshooting	*M*_worst_ < 20%
Tracking accuracy	*e*_error_ < |0.0015| rad
Tracking reference frequency	[0 ÷ 1] rad/s
Input disturbance rejection	|0.05| Nm, [0 ÷ 0.2] rad/s
Output disturbance rejection	|0.5| rad, [0 ÷ 0.5] rad/s
Input current limit	±1.5 A
Voltage limit	±14.8 V
Robust stabilization	
Stable and low-order controllers	K, C

**Table 4:** Allowed region for optimized internal-loop poles ${\tilde{C}}_{\text{in}}(s)$ in a complex plain.

Allowed poles region *D*_in_ for ${\tilde{C}}_{\text{in}}$	
*σ*_*ts*_low_	−0.7
*σ*_*ts*_high_	−4
±*jω*_*tr*_	±*j*10
±*φ*_*ζ*_	±30°

**Table 5:** Allowed region for optimized internal-loop poles ${\tilde{C}}_{\text{out}}(s)$ in a complex plain.

Allowed poles region *D*_out_ for ${\tilde{C}}_{\text{out}}$	
*σ*_*ts*_low_	−0.35
*σ*_*ts*_high_	−9
±*jω*_*tr*_	±*j*14
±*φ*_*ζ*_	±74°

**Table 6:** Parameters of DE-optimization algorithm.

Optimization algorithm parameters	
Number of parents NP	50
Differential weight *F*	0.85
Crossover probability CR	0.92
Mutation strategy	DE/rand/1/bin

**Table 7:** Polynomials $C_{\text{in}},{\tilde{C}}_{\text{in}}$ roots comparisons.

Polynomials	*C*_in_(*s*)	${\tilde{C}}_{\text{in}}(s)$
	−1	−0.944 − *j*0.04
	−1	−0.944 + *j*0.04

**Table 8:** Polynomials $C_{\text{out}},{\tilde{C}}_{\text{out}}$ roots comparisons.

Polynomials	*C*_out_(*s*)	${\tilde{C}}_{\text{out}}(s)$
	−3.4 − *j*12.3	−3.09 − *j*10.2
	−3.4 + *j*12.3	−3.09 + *j*10.2
	−2.5 − *j*3	−2.61 − *j*2.76
	−2.5 + *j*3	−2.61 + *j*2.76
	−1.2	−1.09
	−0.5	−0.48

**Table 9:** Value of robust criteria.

Criteria	||·||_∞_	Value
	||*T*_in_ *W*_*M*_||_*∞*_	0.82
	||*S*_in_ *W*_*I*_||_*∞*_	0.62
	||*T*_*w*′1_||_*∞*_	0.23
	||*S*_out_ *W*_*s*_^−1^||_*∞*_	0.73
	||*W*_*s*_^−1^ *S*_out_ *W*_out_||_*∞*_	0.123
	||*W*_*s*_^−1^ *S*_out_ *W*_noise_||_∞_	0.47
